# Sequence-based identification of *Anopheles* species in eastern Ethiopia

**DOI:** 10.1186/s12936-019-2768-0

**Published:** 2019-04-16

**Authors:** Tamar E. Carter, Solomon Yared, Shantoy Hansel, Karen Lopez, Daniel Janies

**Affiliations:** 1 Department of Biology, Baylor University, Waco, TX USA; 2 Department of Biology, Jigjiga University, Jigjiga, Ethiopia; 3 Department of Bioinformatics and Genomics, University of North Carolina at Charlotte, Charlotte, NC USA

**Keywords:** *Anopheles arabiensis*, Malaria, Phylogenetics, Internal transcribed spacer 2, Cytochrome oxidase subunit I, Horn of Africa

## Abstract

**Background:**

The recent finding of a typically non-African *Anopheles* species in eastern Ethiopia emphasizes the need for detailed species identification and characterization for effective malaria vector surveillance. Molecular approaches increase the accuracy and interoperability of vector surveillance data. To develop effective molecular assays for *Anopheles* identification, it is important to evaluate different genetic loci for the ability to characterize species and population level variation. Here the utility of the internal transcribed spacer 2 (ITS2) and cytochrome oxidase I (COI) loci for detection of *Anopheles* species from understudied regions of eastern Ethiopia was investigated.

**Methods:**

Adult mosquitoes were collected from the Harewe locality (east) and Meki (east central) Ethiopia. PCR and Sanger sequencing were performed for portions of the ITS2 and COI loci. Both NCBI’s Basic Local Alignment Search tool (BLAST) and phylogenetic analysis using a maximum-likelihood approach were performed to identify species of *Anopheles* specimens.

**Results:**

Two species from the east Ethiopian collection, *Anopheles arabiensis* and *Anopheles pretoriensis* were identified. Analyses of ITS2 locus resulted in delineation of both species. In contrast, analysis of COI locus could not be used to delineate *An. arabiensis* from other taxa in *Anopheles gambiae* complex, but could distinguish *An. pretoriensis* sequences from sister taxa.

**Conclusion:**

The lack of clarity from COI sequence analysis highlights potential challenges of species identification within species complexes. These results provide supporting data for the development of molecular assays for delineation of *Anopheles* in east Ethiopia.

**Electronic supplementary material:**

The online version of this article (10.1186/s12936-019-2768-0) contains supplementary material, which is available to authorized users.

## Background

Over 1.5 million cases of malaria were reported in Ethiopia in 2017 [1]. While strides to control the transmission of malaria likely contributed to the reduction in overall mortality and incidence over the last several decades [2], continued understanding of the mosquito vector populations are needed for improved targeted interventions [3]. In east Ethiopia, *Anopheles* species are still being uncovered. A recent study revealed the presence of *Anopheles stephensi*, a malaria vector species typically only seen east of the Red Sea [4]. Historically, the number of malaria cases have been low in this region, but the presence of potential malaria vectors and recent reports of sporadic malaria outbreaks warrant further investigation of vector populations.

Due to the global variation of *Anopheles* species and populations, it is vital to evaluate techniques specific to east Ethiopia to identify various *Anopheles* species [3, 5]. Once a technique is validated the diversity and distribution of various *Anopheles* species can be accurately determined and the proper intervention implemented. In Ethiopia, much of the mosquito surveillance and identification is conducted using mosquito morphology, e.g. [6–9]. Morphological identification can be tedious when processing many specimens and comes with risk for misidentification of species not previously encountered and potentially cryptic species [5]. Genetic analysis can be employed as a high-throughput approach to identify mosquito species. Moreover, because the DNA data are interoperable with previous DNA records and often is linked to rich metadata on location and date of isolation, one can build information about population structure and movement of vector species to improve our understanding of the spatial epidemiology of malaria. Analysis of the nuclear internal transcribed spacer 2 (ITS2) and mitochondrial cytochrome oxidase I (COI, also called CO1, COX1) loci have served as the basis of species identification assays that use allele-specific PCR amplification [10, 11], restriction enzyme digestions [12], or genetic sequencing-based assays [4, 5, 13]. Identifying the correct locus or loci for the basis of species or population-level analysis is important. Previous studies have highlighted how the analysis of the COI gene poses challenges for discriminating between closely related species such as those which belong to a species complex (for review see Beebe et al. [14]). In this study, the ITS2 and the COI loci were sequenced and analysed for species identification in *Anopheles* specimens collected in two sites in east Ethiopia to evaluate the potential of these loci for identification of east Ethiopian *Anopheles*.

## Methods

### Study locations

Mosquito specimens were collected during four collections from two sites, Harewe locality and Meki, in east Ethiopia (Table 1). These regions were selected because malaria cases have been reported there in recent years [15, 16]. The Harewe locality is in the Harari Region at 9°16′N latitude and 42°10′E longitude, 15 km from Harar city. Harewe has a mountainous landscape with an elevation of 1552 m above sea level. A small river runs across the Harewe valley between the mountains and is suspected to be a breeding habitat of *Anopheles* mosquitoes. Meki is a town in east-central Ethiopia, 130 km from Addis Ababa. Meki is located in the East Shewa Zone of the Oromia Region in the middle of the Rift Valley area, at latitude 8°9′N and longitude 38°49′E with an elevation of 1636 m above sea level. Meki has a tropical climate and is surrounded by lakes. Small-scale irrigation is practiced by the community in Meki.

**Table 1:** Specimen collection sites, dates, GPS coordinates, and quantities

Collection site	Date of collection	GPS coordinates	Number of mosquitoes
Harar	November 21, 2016	9.3126°N, 42.1227°E	22
Harar	July 21, 2017	9.3126°N, 42.1227°E	8
Meki	July 28, 2017	8.1552°N, 38.8258°E	31
Harar	August 19, 2017	9.3126°N, 42.1227°E	32
Total			93

### Sample collection

The three collections in the Harewe locality took place in November 2016, July 2017, and August 2017. The Meki collection took place in August 2017. Mosquitoes were collected indoors and outdoors from 6:00 pm to 6:00 am from each selected areas using standard CDC light traps (John W. Hock, Gainesville, FL, USA). Indoor traps were hung from the ceiling or from roof supports at the foot end of beds where people sleep at night. Outdoor collection traps were placed close to breeding habitats and the body of the trap was suspended about 1.5 m from the ground. A total of 16 CDC light traps were deployed for collection of mosquitoes in each study area.

Collected mosquitoes were kept in paper cups and brought to the field laboratory for identification. At the laboratory, mosquitoes were anesthetized with chloroform and all adult mosquitoes were counted and identified, under steromicroscopes, to at least genus level based on a morphological key [17].

### Amplification and sequencing

Molecular analysis was performed on collected *Anopheles* mosquitoes to determine species and characterize the genetic variation within species. Species identification was completed using amplification of two genes: ITS2 and COI. Legs were used as DNA templates for PCR. For ITS2 amplification, PCR amplifications were performed as described previously [4] using the following: primers 5.8S ATCACTCGGCTCGTGGATCG and 28S ATGCTTAAATTTAGGGGGTAGTC for ITS2 [11]. Starting reagents concentrations were as follows: 10 mM for each primer, 2X Promega GoTAQ HotStart master mix (Promega, Madison, Wisconsin), and water for a total reaction volume of 25 µl. PCR amplifications were performed with the following temperature cycling: 95 °C for 2 min, 30 cycles of 95 °C at 30 s, 50 °C at 30 s, 72 °C at 1 min, and final extension of 72 °C at 5 min. The protocol for COI was the same as ITS2 protocol except that the primers used were LCO1490F GGTCAACAAATCATAAAGATATTGG and HCO2198R TAAACTTCAGGGTGACCAAAAAATCA for COI [18]. Temperature cycling for COI PCR was as follows: 95 °C at 1 min, 30 cycles of 95 °C for 30 s, 48 °C for 30 s, 72 °C for 1 min, with a final extension of 72 °C for 10 min. For both ITS2 and COI, eight microliters of PCR product were run on 2% agarose gel for 1 h at 100 V to confirm successful PCR products which were then cleaned using ExoSAP. PCR products were sequenced using Sanger technology with ABI BigDyeTM Terminator v3.1 chemistry (Thermofisher, Santa Clara, CA) according to manufacturer recommendations and run on a 3130 Genetic Analyzer (Thermo Fisher, Santa Clara, CA).

### Sequence analysis for species identification

Sequences were cleaned and analysed using CodonCode Aligner Program V. 6.0.2 (CodonCode Corporation, Centerville, MA). ITS2 and COI sequences from *Anopheles* specimens were submitted as queries to the National Center for Biotechnology Information's (NCBI) web-based Basic Local Alignment Search Tool (BLAST) [19] against the nucleotide collection in Genbank under default parameters [max High-scoring Segment Pairs (HSP) 250, expect threshold 10, word size 28, optimized for highly similar hits, not specific to any organism]. The *Anopheles* subject sequences from NCBI that formed HSP with the queries were identified.

Phylogenetic analyses of ITS2 and COI were employed to search for sister taxon relationships between isolates of *Anopheles* from east Ethiopia and voucher specimens from *Anopheles* with orthologous sequence data stored in NCBI. *Anopheles* sequences from east Ethiopia and closest sequence hits in BLAST that had more than 85% sequence coverage were combined into datasets for COI and ITS2 separately. In some cases, there were multiple sequences from the same location and study. In these instances, only representative sequences were taken from those population sets. Alignments were created with MAFFT version 7 under default parameters [18] and ragged ends were trimmed using Mesquite 3.51 [20]. Phylogenetic relationships with the Ethiopian *Anopheles* sequences and *Anopheles* sequences from NCBI were inferred using RAxML [21] which is based on a maximum likelihood (ML) approach. The GTRGAMMA option that uses GTR model of nucleotide substitution with gamma model of rate of heterogeneity was applied. Both 100 and 1000 replicates were completed with the strategy searching for the heuristically-best-scoring tree and bootstrap analysis in one run. Best scoring trees under ML with bootstrap values from RAxML were viewed and rooted under the outgroup criterion in FigTree [22] for each locus. Outgroups were chosen based on availability of sequence data for each locus, overall coverage, and its use in previous phylogenetic analyses. For the COI analysis, *Anopheles implexus* sequence was used as an outgroup based on sequence availability and use in similar analyses of *Anopheles* species [4]. For the ITS2, a different species, *Anopheles christyi,* was used as an outgroup primarily because *An. implexus* sequence was not available. Compatible *An. christyi* ITS2 sequence was available and this species had been used in a similar analysis [23].

## Results

### ITS2 sequence analysis

The ITS2 sequences were analysed for a subset of samples from each collection from Ethiopia (n = 82). All *Anopheles gambiae* complex specimens from this collection in Ethiopia were identical for ITS2 sequences. When the consensus ITS2 sequence from Ethiopia were searched against NCBI with BLAST, specimens from Ethiopia formed HSP with ~ 99% identity for *Anopheles arabiensis*.

ITS2 sequence data from eight non-*Anopheles gambiae* complex specimens from Ethiopia were generated and all sequences were identical. Based on BLAST against NCBI sequences, these ITS2 sequences from Ethiopia formed HSP with ~ 99% identity for *Anopheles pretoriensis*.

### COI sequence analysis

A subset of the samples from each collection from Ethiopia were chosen for PCR amplification and sequencing of a portion the COI gene (n = 37). Sequences were cleaned and trimmed and submitted as queries to NCBI’s BLAST. Of the 37 sequences from Ethiopian specimens, 29 formed HSP with ~ 99% identity for both *An. arabiensis* and *An. gambiae* sequences in the NCBI database.

These 29 specimens for which COI sequences had ambiguous HSP with respect to species had coinciding ITS2 sequences (see above) that confirmed their identity as *An. arabiensis*. The number of unique COI sequences (haplotypes) was determined. COI sequences with at least 578 bp (n = 20) of readable sequence data revealed 12 different haplotypes.

The remaining eight sequences from Ethiopian specimens formed HSP with NCBI data for within 99% for *An. pretoriensis* sequence vouchers. These eight Ethiopian specimens had coinciding ITS2 data that confirmed their identity as *An. pretoriensis*. Six of these specimens had at least 611 bp of readable sequence and each had a unique COI haplotype.

### Phylogenetic analysis for further species differentiation

**Fig. 1:**
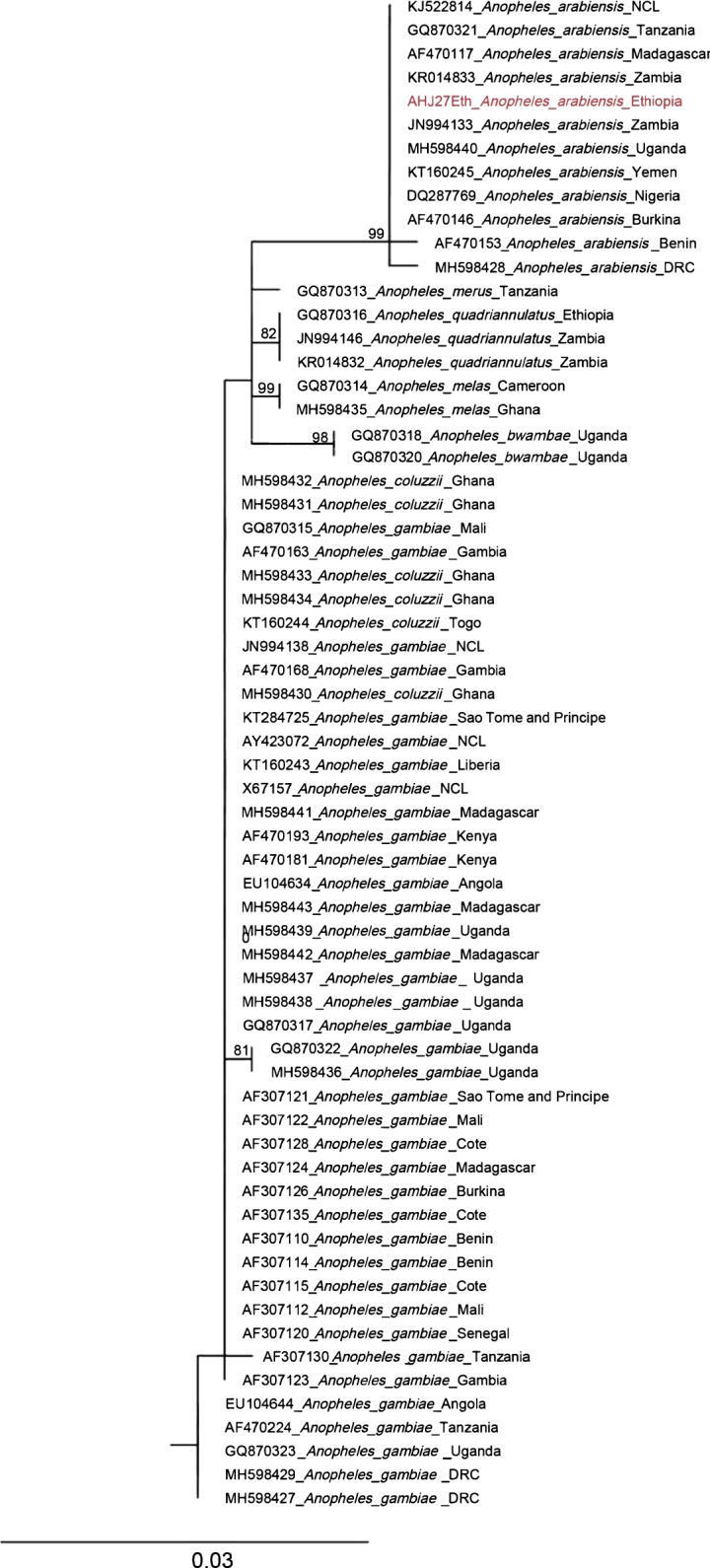
Maximum-likelihood tree of *Anopheles* ITS2 sequences. Analysis based on a 465 base pair sequence of the locus. The taxon in red is a representative specimen collected in Ethiopia from the present study (all Ethiopian ITS2 sequences were identical). Tree includes *An. gambiae* complex sequences taken from NCBI’s Genbank. Bootstrap values 70 and higher are shown. Outgroup (*Anopheles christyi*) not shown. Final ML Optimization Likelihood: − 1110.705351

To confirm the results of the ITS2 BLAST analysis identifying *An. arabiensis* specimens, phylogenetic analysis was performed with the closest hits in Genbank within the *An. gambiae* complex (Additional file 1: Table S1a, Additional file 2). The analysis for 100 and 1000 bootstrap replicates produced similar final ML likelihood scores (both = − 1110.7). Figure 1 shows the tree for the 100 bootstrap replicates. The *An. arabiensis* sequences from NCBI formed a clade that included the Ethiopian specimens with bootstrap support of 99% (Fig. 1). This *An. arabiensis* clade was distinct from all other *An. gambiae complex* species. *Anopheles pretoriensis* and other more distant *Anopheles* taxa could not be included in these analyses due to large deleted regions in the ITS2 of these taxa. However, BLAST analysis was sufficient to determine *An. pretoriensis* identification.

**Fig. 2:**
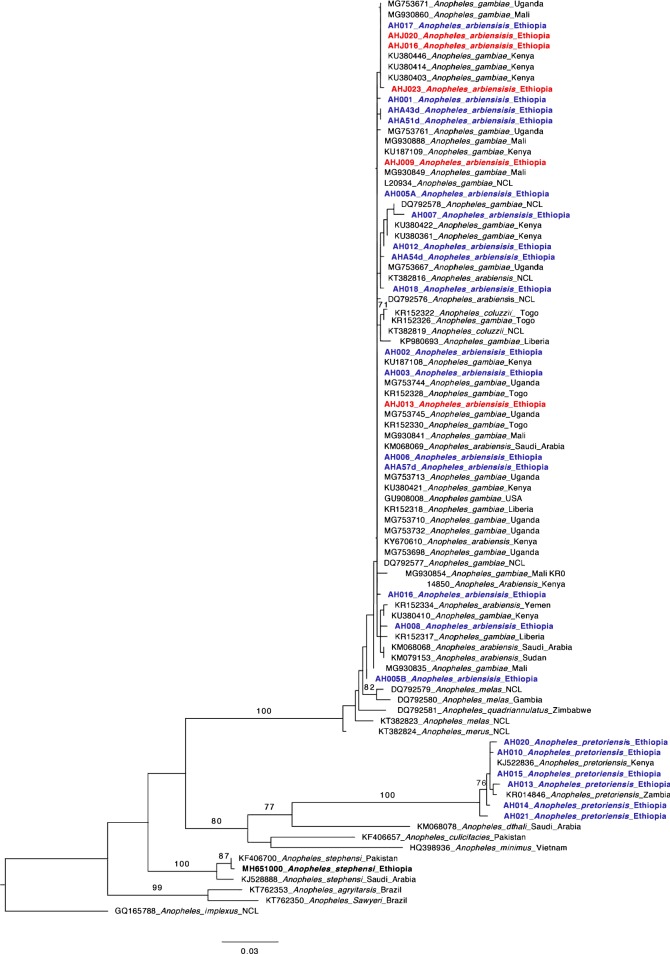
Maximum-likelihood tree of *Anopheles* CO1 sequences. Analysis is based on a 611 base pair sequence of the gene. Taxa in bold are the *Anopheles arabiensis* specimens collected in Ethiopia (species confirmed with ITS2 sequences) from the present study. Taxa in blue were collected in the Harewe locality and red in Meki. Tree includes *An. gambiae* complex and non-*An. gambiae* complex sequences taken from NCBI’s Genbank. Bootstrap values 70 and higher are shown. Final ML Optimization Likelihood: − 2668.816013

Phylogenetic analysis was also completed using the COI sequences. A collection of sequences from the *An. gambiae* complex and representative sequences from other *Anopheles* species (including the Ethiopian *An. stephensi* sequence) were included in the phylogenetic analysis (Additional file 1: Table S1b, Additional file 3). The analysis for 100 and 1000 bootstrap replicates produced similar final ML likelihood scores (both = − 2668.8). Figure 2 shows the tree for the 100 bootstrap replicates. Based on the phylogenetic analysis of COI, the *An. arabiensis* Ethiopian sequences fell within the *An. gambiae* complex clade (Fig. 2, bootstrap = 100) to the exclusion of *An. pretoriensis* and other species outside the *An. gambiae* complex. Within *An. gambiae* complex clade, the *An. arabiensis* and *An. gambiae* sequences could not be differentiated with these data. In addition, no differentiation was observed between the Harewe locality and Meki Ethiopian specimens. The *An. pretoriensis* specimens from NCBI and from Ethiopia formed a clade separate from other *Anopheles* species, with some bootstrap support = 100.

## Discussion

ITS2 and COI sequence variation in *Anopheles* showed different levels of success in identifying *Anopheles* species found in east Ethiopia. Both loci confidently distinguished specimens belonging to the *An. gambiae* complex species from those that do not (i.e. *An. pretoriensis*). ITS2 provides further resolving power to differentiate *An. arabiensis* specimens from other *An. gambiae* complex specimens.

For the COI data, *An. arabiensis* and *An. gambiae* specimens formed a clade that could not be distinguished as species level subclades (Fig. 1). Previous studies have shown similar results with mtDNA [23, 24]. *Anopheles arabiensis* and *An. gambiae* are closely related species so the similarity in sequence may be due to incomplete evolutionary sorting or hybridization between species [14, 23–25] that reduces power to distinguish species. In contrast, COI sequences proved to be very useful for the species identification of *An. stephensi* in Ethiopia [4].

There was interest in whether within species differentiation could be observed for the Ethiopian specimens. While initial sequence analysis showed substantial haplotype variation in the COI locus within the Ethiopian sequences, phylogenetic analysis did not reveal any within-species differentiation, geographic or otherwise for the confirmed *An. arabiensis* sequences. There was some within-species differentiation for *An. pretoriensis* sequences. COI locus revealed some differentiation within the *An. stephensi* grouping [4]. Taken together, these results indicate that COI has some utility related to within species differentiation for some *Anopheles* species, but not for others.

The results presented in this study confirm the presence of *An. pretoriensis* in the Harewe locality. This species has been observed in other parts of Ethiopia including the southwest and northern regions [26, 27]. *Anopheles pretorienis* has not been considered a strong vector of malaria. Indeed, blood meal analysis of two blood-fed *An. pretoriensis* from this study indicated only bovine feeding (data not shown). However, a recent study showed *An. pretoriensis* was positive for *Plasmodium falciparum* in Zambia, suggesting it is important to understand the distribution of this species in Ethiopia as a potential vector [5]. Additionally, subspecies differentiation for one *An. pretoriensis* specimen in the COI analysis was observed (Fig. 2, bootstrap = 74%). Questions remain whether there is significant evolutionary divergence within the *An. pretoriensis* species and if it is associated with vector competence.

These findings have implications for the design of molecular assays to differentiate *Anopheles* species in east Ethiopia. ITS2 has proven to be a more useful sequence-based approach to determine species using simple BLAST analysis. Phylogenetic analysis of the COI can be useful for sequence-based analysis of some *Anopheles* species found in east Ethiopia, but not for members of the *An. gambiae* complex. One approach that may improve molecular species identification would be to combine the two loci into a single analysis. Previous studies have employed multiple loci [13, 24] and require the availability of genomic or coordinating database sequences for both loci from the same specimens representing relevant species and populations. If such sequence data are available, phylogenetic analysis that incorporates genes with various rates of evolution often provides better insight into both between and within species diversity.

## Conclusion

In conclusion, ITS2 and COI vary in their ability to delineate *Anopheles* species. The results of the COI analysis of *An. arabiensis* specimens revealed the potential challenge of using just that locus for molecular species identification of within species complexes. The results of this study contribute to development of molecular assays for *Anopheles* species identification in east Ethiopia.

## Additional files

**Additional file 1.** List of sequences from NCBI database used in phylogenetic analysis.

**Additional file 2.** ITS2 Sequence alignment including Ethiopian and NCBI Genbank sequences.

**Additional file 3.** COI Sequence alignment including Ethiopian and NCBI Genbank sequences.

## Abbreviations

BLAST: Basic Local Alignment Search Tool
COI: cytochrome c oxidase subunit 1 gene
DNA: deoxyribonucleic acid
HSP: high-scoring segment pairs
ITS2: internal transcribed spacer 2 region
NCBI: National Center of Biotechnology Information
PCR: polymerase chain reaction
